# HSCs transdifferentiate primarily to pneumonocytes in radiation-induced lung damage repair

**DOI:** 10.18632/aging.202644

**Published:** 2021-03-03

**Authors:** Lei Li, Suping Zhang, Chaorong Ge, Li Ji, Yaqi Lv, Chen Zhao, Li Xu, Jingyi Zhang, Chenglin Song, Jianing Chen, Wen Wei, Yixuan Fang, Na Yuan, Jianrong Wang

**Affiliations:** 1 Hematology Center of Cyrus Tang Medical Institute, Soochow University School of Medicine, Suzhou 215123, China; 2 National Clinical Research Center for Hematologic Diseases, Collaborative Innovation Center of Hematology, Jiangsu Institute of Hematology, Institute of Blood and Marrow Transplantation, Department of Hematology, The First Affiliated Hospital of Soochow University, Suzhou 215006, China; 3 State Key Laboratory of Radiation Medicine and Protection, Soochow University School of Medicine, Suzhou 215123, China; 4 Department of Hematopoietic Engineering, Susky Life SciTech (Suzhou) Co., Ltd., Suzhou 215124, China

**Keywords:** HSC, transdifferentiation, pneumonocytes, radiation

## Abstract

Accumulative radiation exposure leads to hematopoietic or tissue aging. Whether hematopoietic stem cells (HSCs) are involved in lung damage repair in response to radiation remains controversial. The aim of this study is to identify if HSC can transdifferentiate to pneumonocytes for radiation-induced damage repair. To this end, HSCs from male Rosa^mT/mG^ mice were isolated by fluorescence-activated cell sorting (FACS) and transplanted into lethally irradiated female CD45.1 mice. 4 months after transplantation, transplanted HSC was shown to repair the radiation-induced tissue damage, and donor-derived tdTomato (phycoerythrin, PE) red fluorescence cells and *Ddx3y* representing Y chromosome were detected exclusively in female recipient lung epithelial and endothelial cells. Co-localization of donor-derived cells and recipient lung tissue cells were observed by laser confocal microscopy and image flow cytometry. Furthermore, the results showed HSC transplantation replenished radiation-induced lung HSC depletion and the PE positive repaired lung epithelial cells were identified as donor HSC origin. The above data suggest that donor HSC may migrate to the injured lung of the recipient and some of them can be transdifferentiated to pneumonocytes to repair the injury caused by radiation.

## INTRODUCTION

Exposure to radiation is an inevitable event in human growth, development, and physiological aging. In addition to nuclear accidents, nuclear leakage and nuclear war, the space radiation following development of aerospace projects, medical radiation or tumor radiotherapy in daily life and radiation sources applied in consumer goods, all have different degrees of radioactivity [1–3]. Irradiation is extremely harmful or lethal to human health, and it also showed that accumulative radiation exposure leads to accelerated hematopoietic aging [4–5] or tissue aging [6–8]. Therefore, enhancement of the body resistance to radiation and improvement of treatment strategies post-radiation have been got great attention. Bone marrow transplantation (BMT) is one of the major choices in saving life and repairing the damaged organs or tissues particularly in high-dose radiation, and there is a tremendous amount of effort in BMT studies in human or animal system [3, 9–11]. Nevertheless, the specific type of contributing cells and their underlying mechanism in tissue repair in response to radiation is not fully understood. Bone marrow (BM) harbors hematopoietic stem cells (HSCs), which differentiate into every type of mature blood cells [12]; endothelial cell progenitors; and marrow stromal cells, also called mesenchymal stem cells (MSCs), which can differentiate into mature cells of multiple mesenchymal tissues including fat, bone, and cartilage [13].

Bone marrow-derived cells were widely studied in their repair abilities responding to different stimuli or stress, in which the plasticity or transdifferentiation was proposed as the mechanism for damage repair, but controversy on the role of HSCs remains. The pioneering transdifferentiation study of HSC from Dr. Krause’s group [14], showed that HSC (stained with PKH26^+^ Fr25Lin^-^) from BM was capable of giving rise to multi-organ and multi-lineage engraftment in lethally irradiated mice, including lung, intestine, stomach, skin, bile, et al, especially to lung tissue cells [15–17]. In later studies, a series of similar conclusions were drawn that bone marrow stem cells possess the ability to transdifferentiate to non-hematopoietic tissue cells, such as endothelial precursors [18], hepatic cells [19], intestine cells [20], brain microglia and macroglia [21], skeletal muscle cells [22] and cardiac muscle cells [23, 24]. Notably, most of these studies used BM-derived cells as donor cells, not HSCs alone. On the other hand, opposing opinions were also supported by evidence suggesting that HSCs are incapable of transdifferentiating to non-hematopoietic cells, especially cardiac muscle cells, represented by groups from Dr. Weissman and Dr. Murry [25–27]. There are several aspects that might cause the controversy: purity in donor cell population, reliability in organ damage models, and detection methods. Therefore, further studies are warranted to determine the importance and efficiency in transdifferentiation of HSCs in response to radiation insult. In particular, it is essential to address the dispute by optimizing detection techniques, appropriate donor mouse model and specific HSC purification, suitable recipient mice, and valid controls. The genetically modified Rosa^mT/mG^ mice [28] offer a new opportunity to track the fate of HSCs. Almost all the HSCs from the mice express high-intensity red fluorescence of tdTotamo. So, in this study, we used Rosa^mT/mG^ mice for HSC donors to explore HSC transdifferentiation ability in radiation-induced tissue damage.

## RESULTS

### HSC transplantation repaired the radiation induced lung damage

**Figure 1:**
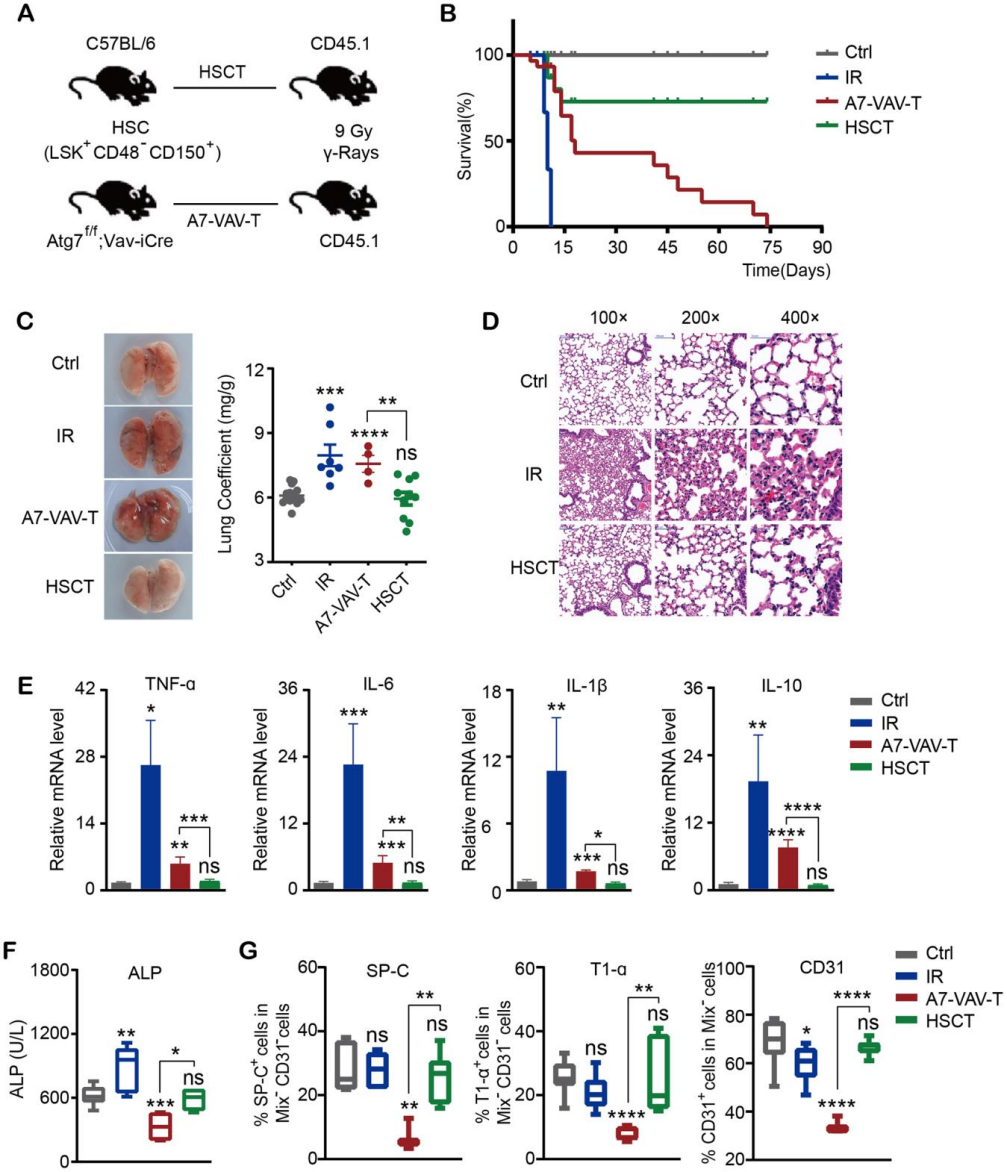
**HSC transplantation repaired the radiation-induced lung damage.** (**A**) Transplantation scheme of HSC (CD48^-^CD150^+^Lin^-^Sca-1^+^c-Kit^+^) from normal C57BL/6 mice and Atg7^-/-^ mice (Atg7^f/f^;Vav-iCre mice) into ^60^Co γ ray 9 Gy lethally irradiated CD45.1 mice. (**B**) The survival time of mice in each group, including Ctrl (Control non-irradiated mice), IR (Mice irradiated with lethal dose of 9 Gy), HSCT (Transplantation of HSC from normal mice), A7-VAV-T (Transplantation of HSC from Atg7^f/f^;Vav-iCre mice). N≥10. (**C**) The lung appearance and coefficient in each group. (**D**) Lung HE pathological alteration in mice, HSCT alleviated radiation-induced pulmonary inflammatory response. (**E**) The inflammatory factors including TNF-α, IL-6, IL-1β, IL-10 expression in each group. (**F**) ALP indicating lung vascular permeability activity in each group. (**G**) Lung epithelial cells (represented by staining of SP-C and T1-α) and endothelial cells (represented by staining of CD31) percentage in each group. N≥4 in C, D, E, F, G. *: p<0.05; **:p<0.01; ***: p<0.001; ****: p<0.0001.

To examine if HSC transplantation (HSCT) could repair the radiation-induced tissue damage, HSCs (CD48^-^CD150^+^Lin^-^Sca-1^+^c-Kit^+^) from normal C57 BL/6 mice and Atg7^-/-^ mice (Atg7^f/f^;Vav-iCre mice, in which the HSC function was severely diminished) [29, 30] were transplanted into ^60^Co γ ray 9 Gy lethally irradiated CD45.1 mice (Figure 1A). The survival time of mice irradiated with lethal dose of 9 Gy was 8-11 days. Transplantation of HSCs from normal mice (HSCT) could save the lives of mice irradiated with lethal dose. But HSCs from Atg7^f/f^;Vav-Cre mice (A7-VAV-T) couldn’t save the lives of irradiated mice, most of which died at about 2 months post transplantation (Figure 1B), indicating that normal HSCs could save the lives of lethally irradiated mice. The lung coefficient in irradiation (IR) group increased as compared to the control group; HSCT returned to normal, but A7-Vav-T group was still increased, like the IR group (Figure 1C). 9 Gy IR induced lung pathological alteration in mice, including smaller alveolar cavity, thickening alveolar septal, inflammatory cell infiltration, bronchial epithelial cell structure destruction and hemorrhage observed in the HE pathological section, while HSCT could alleviate radiation-induced pulmonary inflammatory response, most of which returned to normal (Figure 1D). The inflammatory factors including TNF-α, IL-6, IL-1β, and IL-10 increased significantly in IR group, HSCT reduced this damage; In contrast, these factors remained high in A7-VAV-T group (Figure 1E). ALP (Alkaline phosphatase, assessing lung vascular permeability after radiation [31]) was increased in IR group and returned to normal in HSCT group, but it was getting worse in A7-Vav-T group (Figure 1F). The lung epithelial cells (represented by staining of SP-C and T1-α) and endothelial cells (represented by staining of CD31) decreased significantly in A7-VAV-T group as compared to the control, but returned to normal in HSCT group (Figure 1G). HSCT also repaired other tissue damage induced by radiation (Supplementary Figure 2A–2H). Together, these data suggested that normal HSC could repair the radiation- induced tissue damage.

### Male donor-derived red fluorescence cells and Y chromosome were detected in female recipient lung tissue cells

**Figure 2:**
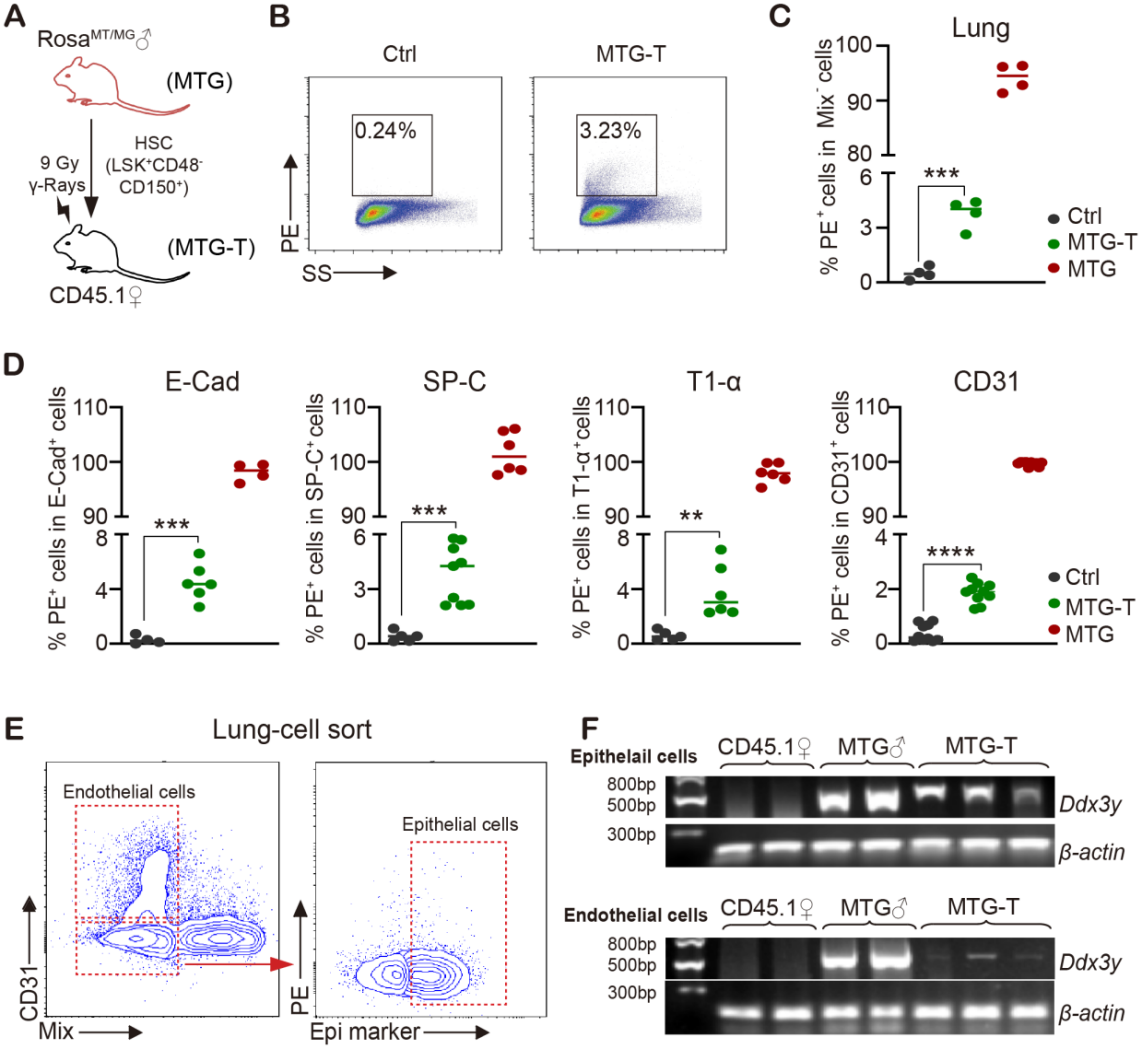
**Male donor-derived PE red fluorescence cells and Y chromosome were detected in female recipient lung tissue cells.** (**A**) HSC from male Rosa^mT/mG^ mice were sorted and transplanted into lethally irradiated female CD45.1 mice. (**B**) Flow cytometry chart of PE (tdTomato) fluorescence in lung tissue cells with exclusion of blood cells including lineage (CD3, CD8, B220, Gr-1, TER119), macrophage (CD11b, F4/80), megakaryocyte (CD41/CD61) and CD45 (marked as Mix). (**C**) Statistical analysis of PE positive percentage in lung tissue cells. (**D**) PE percentage in lung epithelial cells (E-Cadherin, SP-C and T1-α) and endothelial cells (CD31) (after exclusion of blood cells). (**E**) For *Ddx3y* detection, lung epithelial cells (Epithelial marker^+^/blood Mix marker^-^/CD31^-^) and endothelial cells (blood Mix marker^-^/CD31^+^) were sorted. (**F**) Representative images of *Ddx3y* expression by amplification with PCR and detection by nucleic acid electrophoresis. N≥4 in (**C**, **D**, **E**, **F**). **:p<0.01; ***: p<0.001; ****: p<0.0001.

To test whether donor derived cells are present in recipient tissue cells, HSCs from male Rosa^mT/mG^ mice were sorted and transplanted into lethally irradiated female CD45.1 mice (Figure 2A). 4 months after HSCT, the recipient mice were sacrificed, tdTomato/PE red fluorescence of donor mice and *Ddx3y* representing Y chromosome were detected in recipient mice. No-irradiated CD45.1 mice were used as negative control (marked as Ctrl); Rosa^mT/mG^ mice were used as positive control (MTG); transplanted mice were marked as MTG-T.

Exclusion of blood cells was performed by cardiac perfusion and blood cell marker staining. Among the non-hematological tissues, lung tissue cells possessed the highest PE positive percentage of 3.23% (Figure 2B, 2C). With subsequent PE test in lung epithelial cells (E-Cadherin, SP-C and T1-α) and endothelial cells (CD31), it also had around 2%-5% positive PE percentage in different cells (after exclusion of contaminated blood cells) (Figure 2D). For *Ddx3y* detection, lung epithelial cells (Epithelial marker^+^/blood Mix FITC^-^/CD31^-^) and endothelial cells (blood Mix FITC^-^/CD31^+^) were sorted (Figure 2E), DNA of these cells was extracted and then *Ddx3y* was amplified with quantitative PCR and detected by nucleic acid electrophoresis. The results showed that *Ddx3y* was expressed in the female recipient lung epithelial cells and it could also be detected in some endothelial cells of the female recipient mice (Figure 2F). PE red fluorescence could also be detected in the liver and intestine tissue cells to some extent, however, not in heart and kidney cells (Supplementary Figure 2I); but *Ddx3y* could not be tested in any kinds of these tissues (Supplementary Figure 2J).

In order to examine if HSC transdifferentiation occurred in non-radiation setting, we transplanted HSC of Rosa^mT/mG^ mice into non-irradiated CD45.1 mice, and no PE red fluorescence was detectable in any kind of tissues of the recipient mice (Data not shown). These data together suggested that under radiation injury, transplanted HSCs repaired the lung injury at least in part through transdifferentiation.

### Co-localization of donor-derived cells and recipient lung tissue cells were observed

To verify HSC transdifferentiation, confocal immuno-fluorescence and imaging flow cytometry were carried out to observe the morphology of the donor derived cells and recipient lung tissue cells.

**Figure 3:**
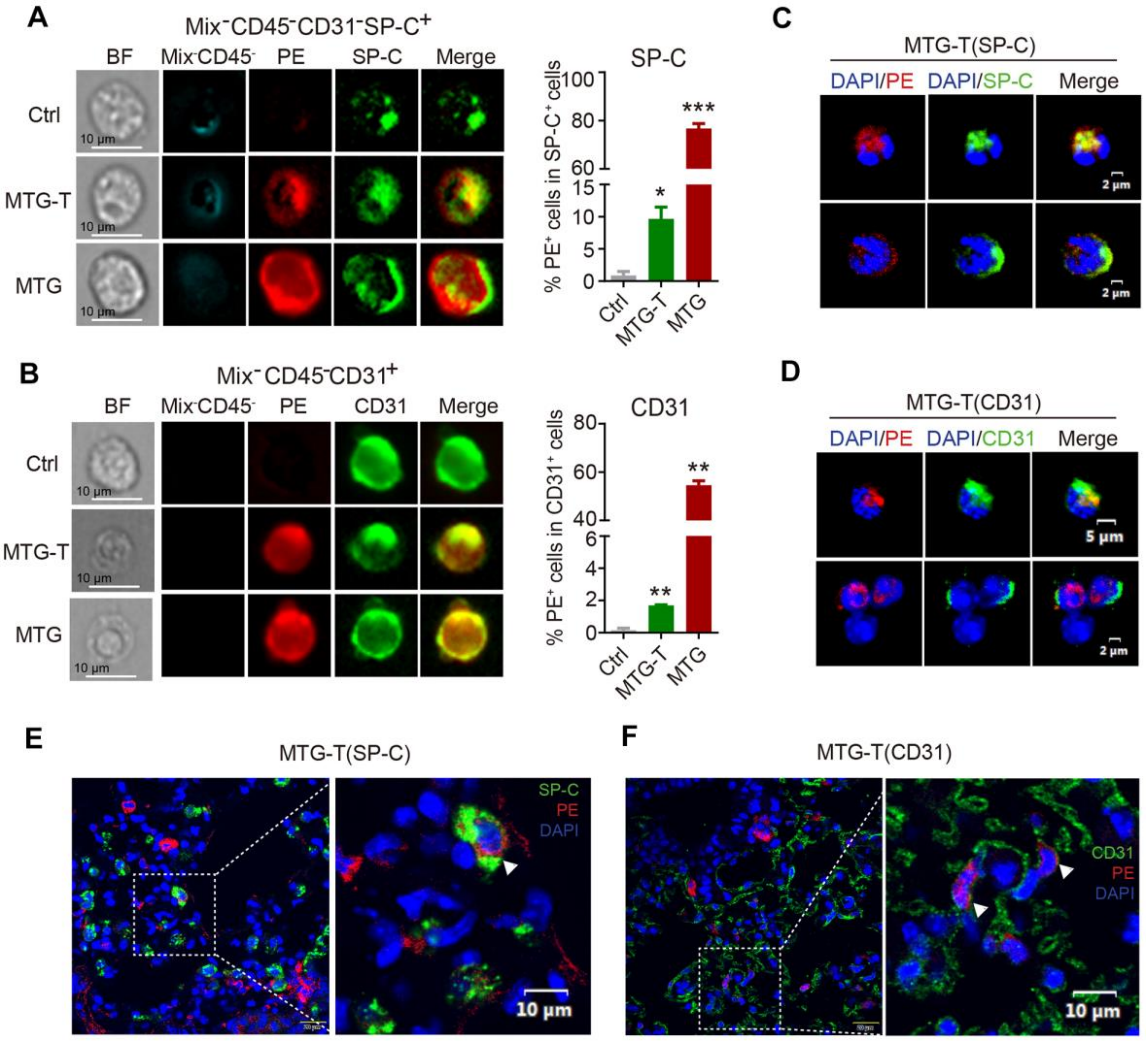
**Co-localization of donor-derived cells and recipient lung tissue cells were observed.** (**A**) The representative images by image flow cytometry results of donor derived PE red fluorescence and the recipient lung epithelial cell marker SP-C and (**B**) endothelial cell marker CD31. The right side showed the quantitative analysis of SP-C with PE, CD31 with PE with exclusion of the blood cells (Blood Mix antibodies and CD45). (**C**, **D**) Representative images of single-cell confocal of PE red fluorescence with lung epithelial cell SP-C and endothelial cell CD31 in sorted single PE positive cells of MTG-T group. (**E**, **F**) Representative images of confocal co-localizaiton of PE red fluorescence with lung epithelial cell marker SP-C, endothelial cell marker CD31 *in situ* lung section of MTG-T group, DAPI was stained to characterize the nucleus.

The image flow cytometry results showed that in MTG-T group, after exclusion of the blood cells, the PE red fluorescence from donor and the recipient lung epithelial cell marker SP-C or endothelial cell marker CD31 were co-localized, with quantitative analysis showing 10% co-localization of SP-C with PE (Figure 3A), and 2% co-localization of CD31 with PE (Figure 3B). The PE positive cells in the lung were sorted, fixed on the slides and stained with lung specific marker. This single cell confocal microscope result also showed co-localization of PE red fluorescence with lung epithelial and endothelial cells (Figure 3C, 3D). For the *in situ* lung section, lung epithelial cell marker SP-C, E-cadherin, pan-Keratin, and endothelial cell marker CD31 were stained, and co-localization of PE red fluorescence with these markers were observed by confocal microscopy, with DAPI staining to characterize the nucleus. It showed co-localization or co-expression of PE and SP-C (Figure 3E), PE and CD31 (Figure 3F), PE and T1-α (Supplementary Figure 3A), PE and P-keratin (Supplementary Figure 3B), PE and E-Cadherin (Supplementary Figure 3C). These data suggested that in morphology, the donor-derived HSC cells could be transformed to recipient lung tissue cells. Moreover, the image flow cytometry and single-cell confocal microscope were both at a single cell level, the images showed that the transformed PE positive cells were all one nucleus, no division or fusion shape, which in somewhat excluded cell fusion.

### HSC transplantation replenished radiation-induced lung HSC depletion and the repaired epithelial cells were of HSC donor origin

**Figure 4:**
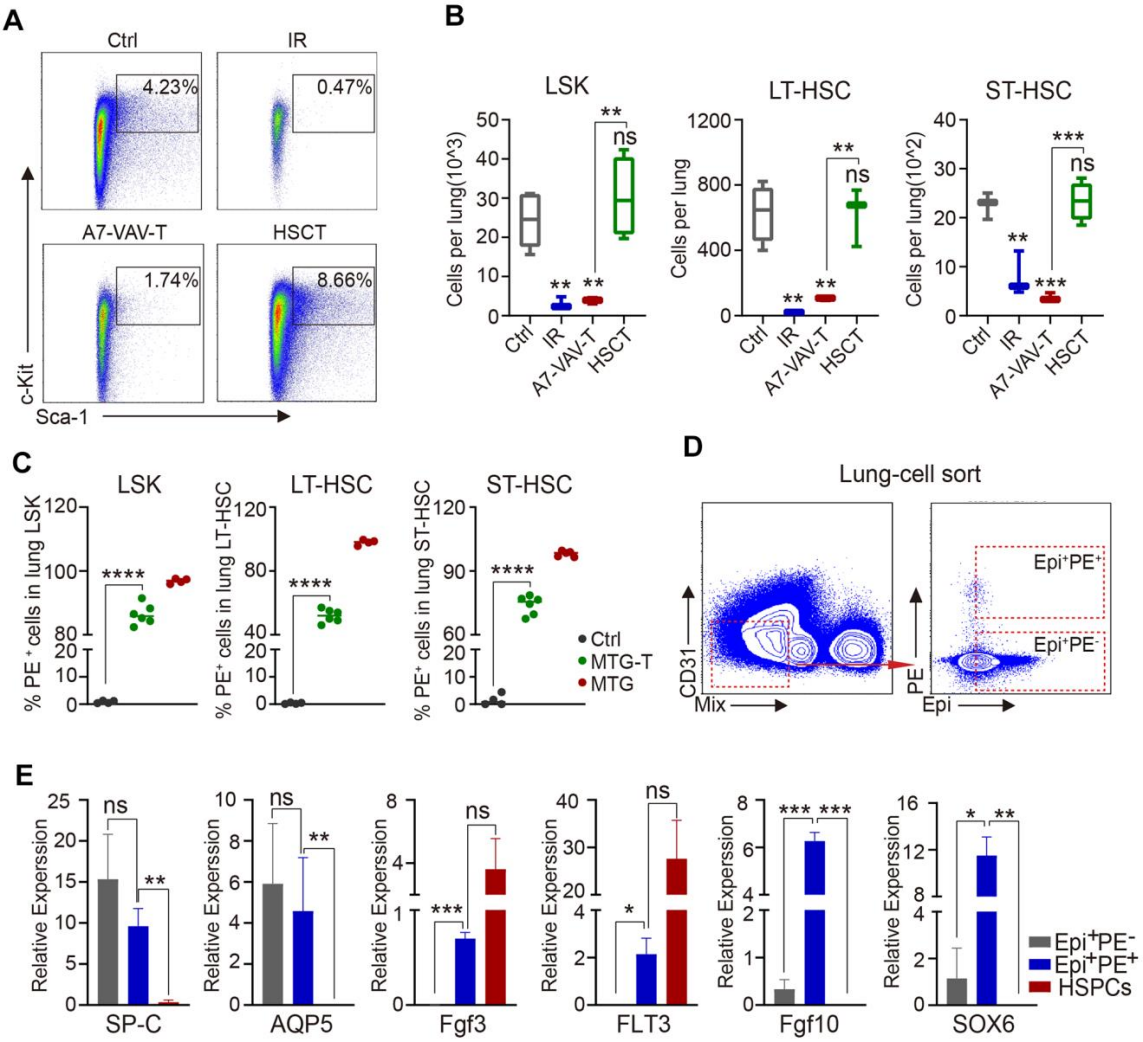
**HSC transplantation replenished radiation-induced lung HSC depletion and the repaired epithelial cells were of donor origin.** (**A**) Flow cytometry chart of HSPC residency in the lung cells of each group, including Ctrl, IR, A7-VAV-T, HSCT. (**B**) HSPC and HSC cell number in the lung in each group, HSCT restored the HSPC and HSC (LT-HSC and ST-HSC), but A7-VAV-T can’t restore the injured HSCs in irradiated lung. (**C**) PE red fluorescence of the HSPCs and HSCs in the lung of Ctrl, MTG-T and MTG group. (**D**) To investigate the function or gene expression in the donor-derived lung cells, the Epi^+^PE^+^, Epi^+^PE^-^ cells in the MTG-T lung were sorted after exclusion of blood Mix cells, Epi markers include E-Cadherin, SP-C and T1-α, and HSPCs from donors were used as control for the experiment. (**E**) Different gene expression including SP-C (alveolar type II epithelial cell marker) and AQP-5 (alveolar type I epithelial cell marker), Fgf3 and FLT3 (hematopoietic stem cell specific genes), Fgf10 and SOX6 (repair genes related) in Epi^+^PE^+^, Epi^+^PE^-^ cells from recipients and HSPC cells from the donors. N≥4. *: p<0.05; **:p<0.01; ***: p<0.001; ****: p<0.0001.

To explore how the BM HSCs repair the injured lung, HSPC (stained against LSK) and HSC residency in the lung were measured by flow cytometry. HSPC and HSC percentage and cell number in the lung all decreased significantly in irradiated group (IR). HSCT restored the HSPCs and HSCs, including LT-HSCs and ST-HSCs, but A7-VAV-T could not restore the injured HSCs in the irradiated lung (Figure 4A, 4B). To determine the source of these restored HSCs, the PE red fluorescence of the HSPCs and HSCs in the lung of MTG-T mice was measured, and it showed that most of the restored HSCs were donor-origin (Figure 4C). To investigate the function or gene expression in these donor-derived lung cells, the Epi^+^PE^+^ and Epi^+^PE^-^ cells in the MTG-T lung were sorted (Figure 4D), and HSPCs from donor were used as control. The Epi^+^PE^+^ and Epi^+^PE^-^ cells both expressed SP-C (alveolar type II epithelial cell marker) and AQP-5 (alveolar type I epithelial cell marker), which confirmed the epithelial characteristics of the PE^+^ cells in the lung (Figure 4E). Fgf3 and FLT3 (HSC specific genes, [32, 33]) were expressed in the Epi^+^PE^+^ cells, suggesting that the cells were derived from donor HSCs. Fgf10 (Fibroblast growth factor 10) and SOX6 (SRY-Box Transcription Factor 6), which are involved in damage repair [34, 35], were only expressed in the Epi^+^PE^+^ cells, not in Epi^+^PE^-^ cells, indicating that these PE^+^ epithelial cells were repaired cells (Figure 4E). These data further suggested that upon irradiation, HSCs from donor BM may migrate to the lungs of the recipients and some of them can be transdifferentiated into lung epithelial cells to repair the lung injury.

## DISCUSSION

Lung is one of the several moderately radio-sensitive organs, and lung epithelial cells are fairly sensitive to radiation rays [36, 37]. Radiation-induced lung injury is a common complication of acute radiation syndrome and chest tumor radiotherapy. Its occurrence is often related to the production of reactive oxygen species (ROS), enhancement of signal transduction such as tumor necrosis factor α, transforming growth factor β and other cytokines. These radiation-triggered factors can result in early radiation pneumonitis [38] and late radiation-induced pulmonary fibrosis [39], in which pulmonary fibrosis is often associated with lung aging [40]. In radiation- induced aging, some small molecules such as ABT263 and piperlongumine have been explored for novel senolytic agents in aged HSCs of mice or *in vitro* lung fibroblast cells [41–43]. However, for high dose radiation diseases, reduction of the acute radiation pneumonitis and late pulmonary fibrosis is of great importance, especially in the current world facing potential nuclear risk. Bone marrow transplantation is one of the most important methods for the therapy, which could restore hematopoiesis and enhance the repair of damaged tissues and organs [44]. But which group of cells in bone marrow contribute to the repair and how the repair is achieved are still unclear. Many studies showed bone marrow MSCs retain their multi-lineage differentiation capacity; however, studies have also shown that MSCs actually enhance the progression of lung injuries, suggest that MSCs may have dual effects and that may limit the application of MSCs [45].

Plasticity of HSCs is the ability of HSCs to differentiate into a variety of non-hematopoietic tissues. Transdifferentiation represents the irreversible conversion of cells from one differentiated cell type to another. Since 1998, there have been many exciting discoveries indicating that stem cells derived from the BM can differentiate into mature, non-hematopoietic cells of multiple tissues, including epithelial cells of the lung [46, 47, 14–24], although opposing results were reported by several groups [25–27]. Despite the controversy, these positive results suggest that stem cells derived from the bone marrow may become valuable tools for cell replacement strategies and regenerative medicine in the future. But it needs more accurate methods and models to verify the existence of transdifferentiation.

To track the fate of bone marrow stem cells (BMSCs), researchers usually transplant BMSCs from a donor to a recipient that differs genotypically or phenotypically. To date, the most commonly used donor specific markers are the Y chromosome (in sex-mismatched transplantations) and transgenes [48]. Another approach to identify donor-derived cells is to use normal mice with genetic polymorphisms that can be detected in all daughter cells. The transgenic mice with GFP, EGFP and Rosa^mT/mG^ are mice with genetic fluorescent markers [28, 49, 50]. But the genetic stability and fluorescence strength in the mice might be different, and there might be gene silence occurred. GFP transgenic mice are derived from the CMV enhancer of chicken β-actin promoter, no convincing evidence has shown that all transplanted BM cells or HSCs express high intensity of GFP fluorescence [51]. Likewise, whether the background auto-fluorescence is expressed in the newly differentiated cells is also uncertain, so it is difficult to detect the fluorescence signal in the recipient. However, the high intensity and stable expression of red fluorescence from Rosa^mT/mG^ mice used in our study provides a reliable system. The genetically modified mice Rosa^mT/mG^ with a dual fluorescents brought new opportunity to track the fate of BM stem cells. All the cells from the mice express high intensity red fluorescence of tdTotamo (Supplementary Figure 1). Therefore, in this study Rosa^mT/mG^ mice were used as HSC donor to explore the transdifferentiation repair potential in radiation-induced tissue damage.

As the major and most important stem cells in BM, HSCs are one of the widely studied stem cells. The labeling and purification of HSCs also developed over time. In the 1990s, Weissman et al performed LSK markers to label HSPCs, and later, CD34 and Flk2 were introduced to distinguish long term and short term HSCs [52, 53]. Around 2000s, SLAM family (CD41, CD48 and CD150) was used to enrich the HSCs since SLAM is stable and widely expressed in different mice [54, 55]. Thus, in our study, CD48^-^CD150^+^ LSK were used for purification and sorting of LT-HSCs.

To confirm HSC transdifferentiation, it needs to satisfy one or more of the following criteria: (i) the donor derived cell stains with tissue-specific markers; (ii) the donor derived cell does not stain with a monoclonal antibody to hematopoietic CD45; and (iii) the cell exhibits distinctive morphology, indicative of a differentiated, nonhematopoietic cell fate [25, 56].

With these guidelines, we validated the HSC transdifferentiation from several aspects, including donor derived transgenic tdTomato/PE red fluorescence and genetic marker of Y chromosome in the recipient mice, cell morphology of confocal colocalization between donor cells and recipient cells, with exclusion of the interference of blood cells and cell fusion. First, we validated that HSCs from normal mice repaired the tissue injury induced by irradiation, including saving life of lethally irradiated mice, alleviating radiation-induced inflammatory response and fibrosis, recovering of vascular permeability. With HSC transplantation from male Rosa^mT/mG^ mice to female mice, PE red fluorescence and Y chromosome from donor mice were detected in recipient lung epithelial cells and endothelial cells. The lung epithelial cell marker including E-cadherin, SP-C of alveolar type II cells, T1-α of alveolar type I cells were stained by exclusion of hematopoietic cells (lineage cells, CD45 blood cells, macrophage and megakaryocytes) and endothelial cells (CD31). CD31 of endothelial cells were stained by exclusion of hematopoietic cells and lung epithelial cells [57]. *Ddx3y* representing Y chromosome was detected in the female recipient lung epithelial cells and endothelial cells. The co-localizaiton of donor derived PE fluorescence and recipient lung tissue cells were also observed by confocal microscopy. These data suggested that donor derived HSCs might differentiate to lung epithelial or endothelial cells. To exclude the co-localization by cell fusion, single cell confocal and image flow cytometry were performed. The transdifferentiated donor derived lung epithelial cells were proved to be single cells and single nucleus from the results. Last, the donor-derived epithelial cells were verified from HSC origin and characterized of epithelial cells and repaired cells. These data suggested that HSC transdifferentiated to lung pneumonocytes, which contributes to the repair of the lung injury imposed by radiation.

Controversies about the transdifferentiation of hematopoietic stem cells are mainly resulted from different animal systems and methodologies that researchers applied, such as the type and age of donor mice, the limitations of the analysis methods, and the status of the recipients (the source and degree of injury). The use of older mice may limit the ability of HSC transdifferentiation. Studies have shown that younger mice have stronger differentiation ability than old mice [58]. The purification of HSCs is another critical point. The marker used by Krause's team (which supported transdifferentiation) at that time might not be very accurate (PKH26^+^Fr25Lin^-^ marker) for HSCs [14], so the extensive transdifferentiation phenomenon detected might be false positive. Kotton DN et al used SP cells to mark HSCs, they didn’t observe the reconstitution [59]. Third, the injury of recipients may be an essential point for triggering transdifferentiation. The type and degree of injury may affect the transdifferentiation ability of HSCs, and there might be a threshold injury required for triggering transdifferentiation [60–62]. So donor- derived cells were undetectable in recipient mice without irradiation.

**Figure 5:**
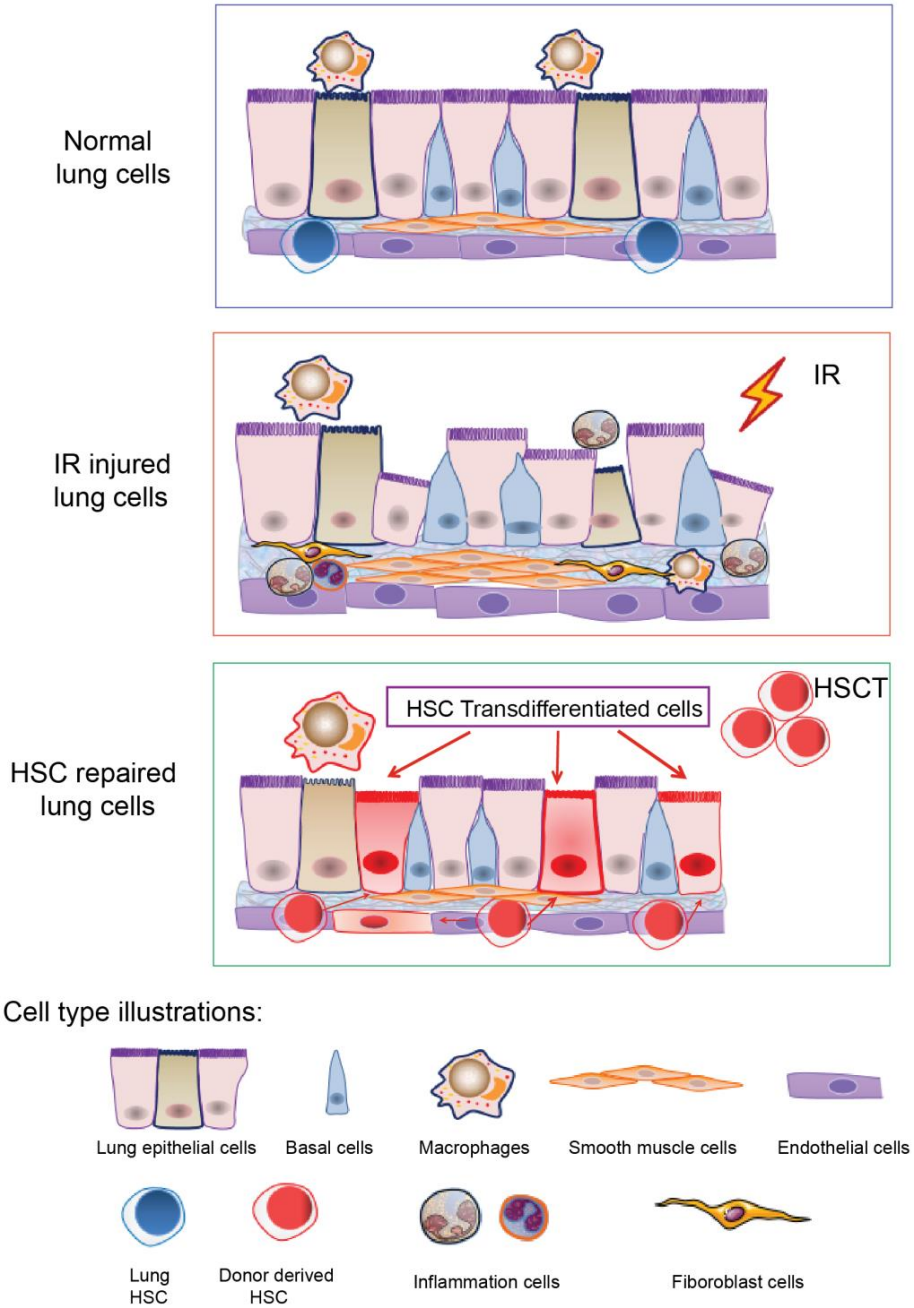
**A cartoon illustrating HSCs transdifferentiation to pneumonocytes for irradiated lung repair.** The cartoon illustrates HSCs transdifferentiate into the lung cells under certain injury such as irradiation. Irradiation induced lung bronchial epithelial cell structure destruction, inflammatory cell infiltration and fibrous hyperplasia and impaired HSC residency in the lung. After HSCT, donor HSCs migrate into the injured lung, and some of them can be transdifferentiated to lung epithelial and endothelial cells to repair the injury imposed by irradiation.

Up to all, we speculate that the transdifferentiation of hematopoietic stem cells may not be a normal physiological phenomenon, but more likely to be one of the repair mechanisms of tissues and organs in the non-physiological injury of the body, and the transdifferentiation may be limited to certain tissues such as the lung tissue that possesses special structure. Lung tissue acts as the barrier of blood gas exchange and provides oxygen and fresh arterial blood for the body. There are abundant capillaries in lung tissue. Recently, the lung has been identified as one of the organs for platelet production and as an organ for storing hematopoietic stem and progenitor cells [63, 64]. So we conjecture that in the case of radiation damage (including hematopoietic and non-hematopoietic damage), after HSC transplantation, the donor HSCs reconstitute the hematopoietic system first, and subsequently, the repaired HSC might enter the injured lung, thereby initiating lung repair cascade. In addition, the increase of lung growth factors (such as Fgf10) and transcription factors (such as SOX6) might promote the transdifferentiation of HSC to lung epithelial or endothelial cells through paracrine or nuclear transcription (Figure 5 illustrates the cartoon of HSCs transdifferentiation to pneumonocytes for irradiated lung repair). Nevertheless, the mechanism responsible for HSC transdifferentiation remains to be resolved in our future study.

In summary, we conclude that donor HSCs from bone marrow may migrate to the injured lung of the recipient, and some of them can be transdifferentiated to lung epithelial and endothelial cells to repair the injury caused by radiation. Future study is still warranted to illustrate the underlying mechanism for HSC transdifferentiation.

## MATERIALS AND METHODS

### Mice and HSC transplantation

C57BL/6, Atg7^f/f^;Vav-iCre, Rosa^mT/mG^ mice were used as donors, CD45.1 mice were used as recipients in this study. Atg7^f/f^;Vav-iCre mice were generated by crossing Atg7^f/f^ mice (kindly from Dr. Komatsu, Japan and breded in our lab) [65, 66] and Vav-iCre mice (purchased from the Jackson Laboratory). Rosa^mT/mG^ mice were kindly from Dr. Yulong He lab in Hematology Center of Cyrus Tang Medical Institue, Soochow University of China. The recipients and donor mice were all 6-8 weeks while experiments. Before HSC transplantation, the recipient CD45.1 mice were irradiated with ^60^Co γ-ray lethal dose of 9 Gy. HSC (CD48^-^CD150^+^LSK) from the donor mice were sorted by FACS (BD FACS Aria III, USA) and a total of 2000 HSC were transplanted into one recipient mouse within 4 hours after irradiation. For lung damage repair detection, C57BL/6 and Atg7^f/f^;Vav-iCre mice were as HSC donors (HSCT and A7-VAV-T), non-irradiated CD45.1 were used as negative control (Ctrl), irradiated CD45.1 mice positive control (IR). For trans-differentiation study, male Rosa^mT/mG^ mice were used as HSC donors (MTG-T), non-irradiated CD45.1 were used as negative control(Ctrl), Rosa^mT/mG^ mice were used as positive control (MTG). All experimental procedures with animals were approved by Soochow University Institutional Animal Care and Use Committee. Reagents used in this study were listed in Supplementary Table 1.

### Lung damage repair detection

For lung damage repair detection, C57BL/6 and Atg7^f/f^; iVav-Cre mice were as HSC donors. Survival of each group (Ctrl, IR, HSCT and A7-VAV-T) was recorded. For other experiments, eight weeks after transplantation (the IR group was 7 days after irradiation), the mice were sacrificed. Lung coefficient was calculated by lung weight to mice weight. The pathological changes of lung in each group were observed by HE staining. RNA of lung tissue was extracted and the relative expression levels of inflammatory factors IL-1β, IL-6, TNF-α, IL-10 in lung tissues of each group were quantified using Q-PCR (LightCycler 480 II, Roche, Switzerland) to measure radiation-induced inflammatory response. Primer sequence were seen in Supplementary Table 2. ALP was measured to assess lung vascular permeability after radiation by biochemical test. Lung tissue cell (SP-C of alveolar type II cells, T1-α of alveolar type I cells, CD31 of endothelial cells) percentage alteration was examined by flow cytometry (Beckman Coulter, USA).

### PE fluorescence detection by flow cytometry

To test whether donor derived cells are present in recipient tissue, flow cytometry was performed to detect the red tdTomato or PE fluorescence in recipient mice. 4 months after HSCT, the recipient mice were sacrificed, cardiac perfusion were performed in mice in order to remove the influence of blood cells in circulation. Bone marrow, heart, liver, lung, kidney, small intestine of mice in each group were taken and digested into single cells by collagenase, dispase II and DNase I incubation in 37° C for 1 h. Antibodies of blood cells (marked as Mix) including lineage (CD3, CD8, B220, Gr-1, TER119), macrophage (CD11b, F4/80), megakaryocyte (CD41/CD61) and CD45 were stained to subsequently exclude the influence of blood cells. After exclusion of these cells, PE (tdTomato) fluorescence in tissue single cells were detected by flow cytometry (Beckman Coulter). PE fluorescence in alveolar type II cells (SP-C), alveolar type I cells (T1-α), other epithelial cells (E-Cadherin), endothelial cells (CD31) was also measured.

### Y chromosome detection

To test whether donor-derived Y chromosome are present in recipient tissue, *Ddx3y* represented Y chromosome was examined by PCR in recipients lung epithelial cells and endothelial cells. Lung epithelial cells (Epithelial marker^+^/blood Mix marker^-^/CD31^-^) and endothelial cells (blood Mix marker^-^/CD31^+^) were sorted by FACS, DNA of these cells were extracted and then *Ddx3y* was amplified with PCR and detected by nucleic acid electrophoresis. Epithelial markers for sorting include SP-C, T1-α and E-Cadherin. Ddx3y primer sequence were seen in Supplementary Table 2.

### Confocal microscope and image flow cytometry

Confocal microscope (Olympus FV1000MPE, Japan) and imaging flow cytometry (Amnis ImageStream MarkII, Merck Millipore, USA) were utilized to analyze the co-expression between donor marker (tdTomato-red fluorescence) and lung epithelial cell markers. For confocal microscope, frozen section of lung tissue was made after the cardiac perfusion of mice. Lung epithelial cell and endothelial cell markers (including Pan-keratin, T1-α, SP-C, E-Cadherin and CD31) conjugated with FITC and DAPI were stained respectively and observed in laser confocal microscope. The co-localization of PE and recipient lung cells were inspected. For image flow cytometry, lung tissue was digested into single cells and stained with lung epithelial and endothelial cell marker (including T1-α, SP-C and CD31) conjugated with APC, DAPI, and exclusion with blood cells (described as above Mix and CD45). The co-localization of PE fluorescence and lung epithelial/endothelial cells were quantified by the image flow wizard software.

### Statistical analysis

Statistical analyses were performed using SPSS version 22.0. The statistical significance of the observed differences was determined by unpaired t tests. Data were expressed as mean ± standard error of the mean (SEM). P<0.05 was considered to indicate a statistically significant difference.

## Supplementary Material

Supplementary Figures

Supplementary Tables
