# Supplementary material for: HSCs transdifferentiate primarily to pneumonocytes in radiation-induced lung damage repair
Source: Aging (Albany NY). 2021 Mar 3;13(6):8335–54. doi: 10.18632/aging.202644 (PMC8034935; doi:10.18632/aging.202644)
Supplement: Supplementary Figures [file aging-13-202644-s001.pdf]

SUPPLEMENTARY FIGURES

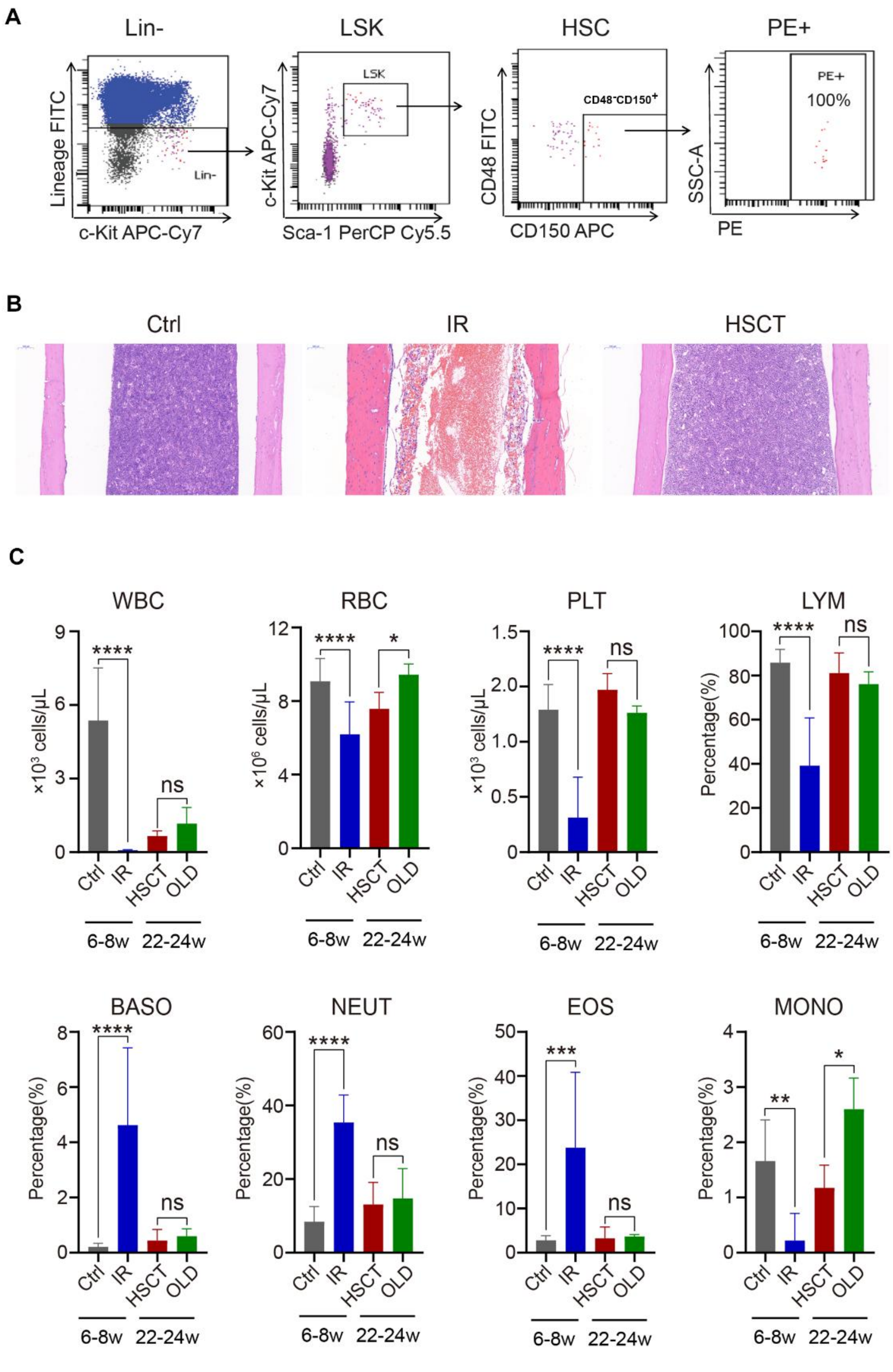

Supplementary Figure 1. PE red fluorescence in donor HSCs and HSCT regenerated the hematopoiesis in the irradiated mice. (**A**) PE red fluorescence in HSC of donor Rosa$^{mT/mG}$ mice. (**B**) HSCT recovered the bone marrow injury induced by irradiation in HE pathology. (**C**) HSCT regenerated the peripheral blood cells including WBC (white blood cells), RBC (red blood cells), PLT (platelets), LYM (lymphocytes), BASO (basophilic granulocytes), NEUT (neutrophile granulocytes), EOS (eosinophilic granulocytes) and MONO (monocytes). N≥4. *: $p<0.05$; **: $p<0.01$; ***: $p<0.001$; ****: $p<0.0001$.

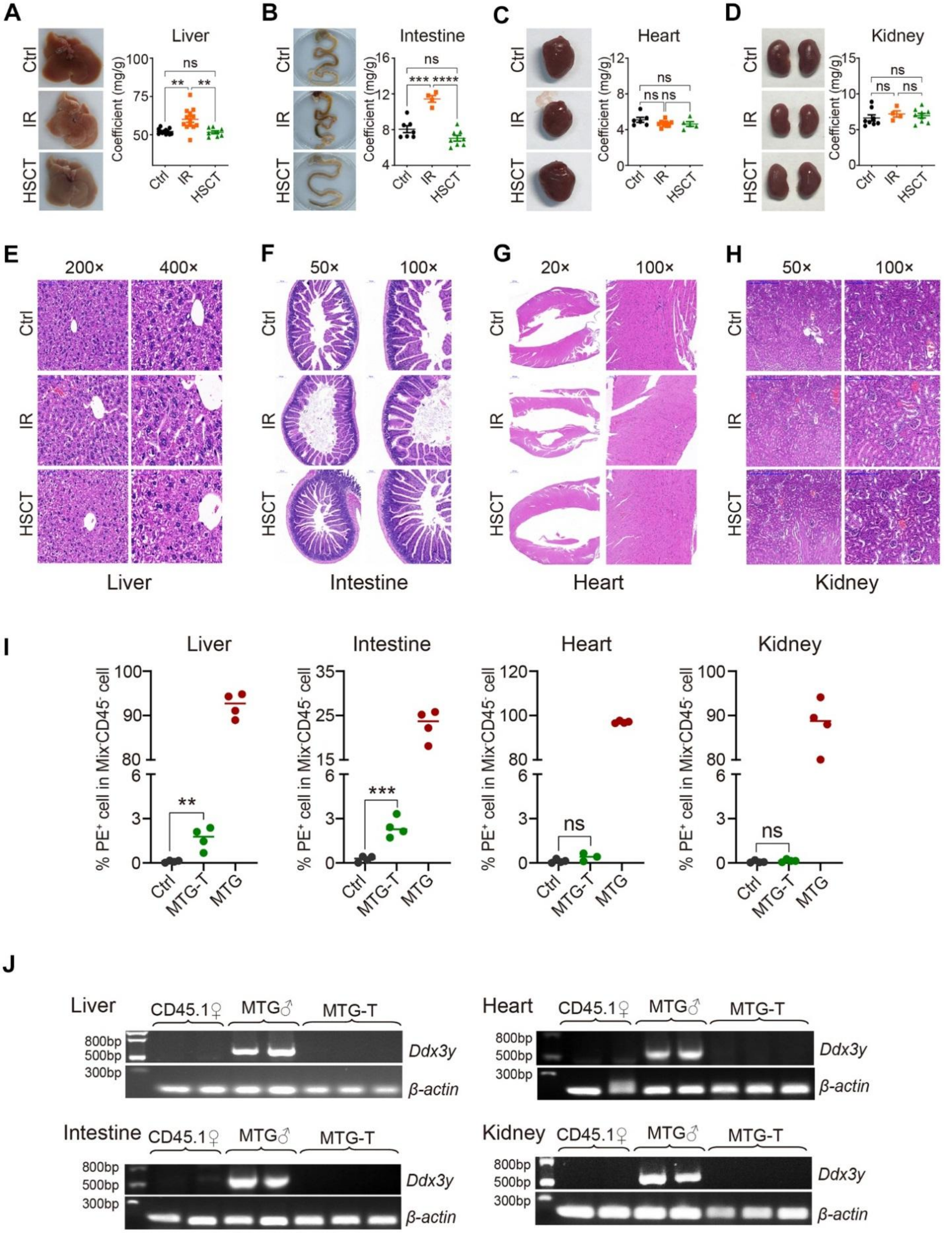

Supplementary Figure 2. HSCT repaired the tissue injury but not through trans-differentiation. (**A**–**D**) The tissue appearance and coefficient including liver, intestine, heart and kidney in each group. (**E**–**H**) The tissue HE pathological section in each group. (**I**) The PE red fluorescence in Blood Mix$^-$ CD45$^-$ tissue cells in each group. (**J**) Representative images of *Ddx3y* expression in each group. N≥4. **:p<0.01; ***: p<0.001; ****: p<0.0001.

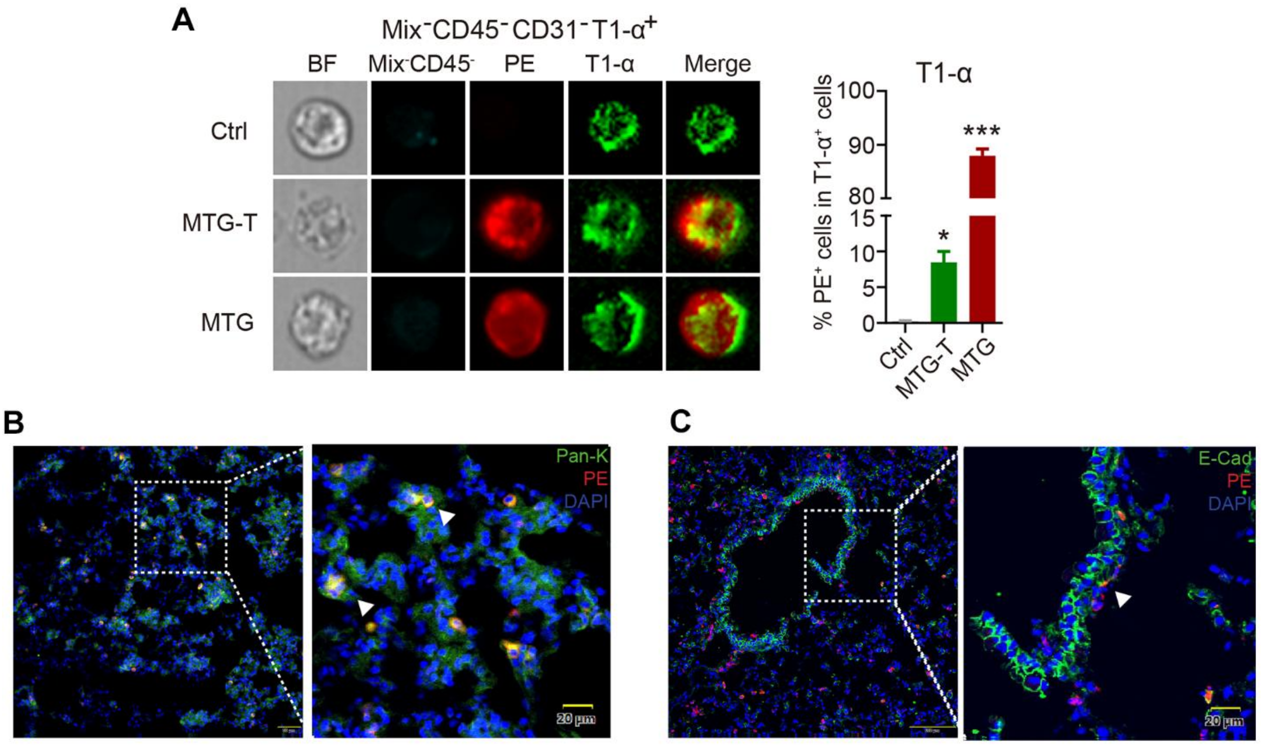

Supplementary Figure 3. Co-localization of donor derived PE fluorescence and recipient lung cells. (**A**) Co-localization of PE and T1-α in recipient Mix-CD45-CD31-T1-α lung cells by image flow cytometry. (**B**) Co-localization of PE and Pan-Keratin in recipient lung tissues by confocal microscope. (**C**) Co-localization of PE and E-Cadherin in recipient lung tissues by confocal microscope. N≥4.
