# Supplementary material for: HSCs transdifferentiate primarily to pneumonocytes in radiation-induced lung damage repair
Source: Aging (Albany NY). 2021 Mar 3;13(6):8335–54. doi: 10.18632/aging.202644 (PMC8034935; doi:10.18632/aging.202644)
Supplement: Supplementary Tables [file aging-13-202644-s002.pdf]

SUPPLEMENTARY TABLES

Supplementary Table 1. Reagents used in this study.

Reagents	Brand company	Catalog No
CD45 PerCP cy5.5	Invitrogen	45045182
CD45 APC/cy7	Invitrogen	47045182
Lineage antibody(CD2,CD3, CD5, CD8a, TER119, Gr-1, B220) FITC	BioLegend	B220:103205; Ter119:116206; CD4:100406; CD8a:100706; CD2:100105; CD3:100204; CD5:100606; Gr-1:108406
CD41 FITC	BioLegend	133904
CD61 FITC	BioLegend	11061182
CD11b FITC	BioLegend	101206
F4/80 FITC	BioLegend	123108
CD48 FITC	Invitrogen	11048182
CD150 APC	BioLegend	115918
Sca-1 PerCP cy5.5	Invitrogen	45598182
c-Kit APC/cy7	Invitrogen	47117282
SP-C APC	Bioss	BS10067R
T1a APC	Bioss	BS10673R
E-Candherin APC	BioLegend	147308
CD31 APC/cy7	BioLegend	102528
PI	BD Biosience	51-66211E
CD48 APC	BioLegend	103412
CD150 BV421	BD Biosience	566298
SP-C	Abcam	ab211326
CD31	Abcam	ab28364
E-Cad	Abcam	ab11512
Goat anti rabbit(AF488)	Abcam	ab150077
Pan-Keratin	Cell Signaling Technology	4523s
DAPI	Beyotime	C1002
Sca-1 AF700	Invitrogen	56598182
Collagenase	Sigma	C2674
DNase I	Sigma	D5025
Dispase II	Sigma	D4693
MicroElute RNA Kit	Omega	R6831

Supplementary Table 2. Primers used in this study.

Primer target	Forward sequences	Reverse sequences
IL-1β	GGAGAACCAAGCAACGACAAAATA	TGGGGAACTCTGCAGACTCAAAC
IL-6	TAGTCCTTCCTACCCCAATTTCC	TTGGTCCTTAGCCACTCCTTC
IL-10	CCAAGCCTTATCGGAAATGA	TTTTCACAGGGGAGAAATCG
TNF-α	CCACCACGCTCTTCTGTCTAC	AGGGTCTGGGCCATAGAACT
SP-C	ATGGAGAGTCCACCGGATTAC	ACCACGATGAGAAGGCGTTTG
AQP5	TCTTGTGGGGATCTACTTCACC	TGAGAGGGGCTGAACCGAT
Fgf3	GCGCCTATAGCATCCTGGAGATTAC	GATCCGTTCCACAAACTCACACTCT
FLT3	GAGCGACTCCAGCTACGTC	ACCCAGTGAAAATATCTCCCAGA
Fgf10	GAGAAGGCTGTTCTCCTTCACCAAG	CTTTGACGGCAACAACTCCGATTTC
SOX6	AATGCACAAACCTCACTCT	AGGTAGACGTATTTCGGAAGGA
GAPDH	AGCTTGTCATCAACGGGAAG	TTTGATGTTAGTGGGGTCTCG
β-actin	TCGTGCGTGACATCAAAGAGA	GAACCGCTCGTTGCCAATA
Ddx3y	CCAATAGCAGCCGAAGTAGTGGTAG	TTAGGGTACAACCAAGCAGGAAGTG
